# Cycles, sources, and sinks: Conceptualizing how phosphate balance modulates carbon flux using yeast metabolic networks

**DOI:** 10.7554/eLife.63341

**Published:** 2021-02-05

**Authors:** Ritu Gupta, Sunil Laxman

**Affiliations:** Institute for Stem Cell Science and Regenerative Medicine (inStem)BangaloreIndia; University of GenevaSwitzerland; Max Planck Institute for Developmental BiologyGermany

**Keywords:** phosphate, mass action, carbon metabolism, metabolic flux, gene expression, metabolic networks

## Abstract

Phosphates are ubiquitous molecules that enable critical intracellular biochemical reactions. Therefore, cells have elaborate responses to phosphate limitation. Our understanding of long-term transcriptional responses to phosphate limitation is extensive. Contrastingly, a systems-level perspective presenting unifying biochemical concepts to interpret how phosphate balance is critically coupled to (and controls) metabolic information flow is missing. To conceptualize such processes, utilizing yeast metabolic networks we categorize phosphates utilized in metabolism into cycles, sources and sinks. Through this, we identify metabolic reactions leading to putative phosphate sources or sinks. With this conceptualization, we illustrate how mass action driven flux towards sources and sinks enable cells to manage phosphate availability during transient/immediate phosphate limitations. We thereby identify how intracellular phosphate availability will predictably alter specific nodes in carbon metabolism, and determine signature cellular metabolic states. Finally, we identify a need to understand intracellular phosphate pools, in order to address mechanisms of phosphate regulation and restoration.

## Introduction

Phosphorus, like other essential chemical elements – carbon, nitrogen, oxygen, and sulfur, is central to life. Inside cells, phosphorus is found in the form of salts and esters of phosphoric acid (orthophosphates, or simply Pi), and is widely present in a range of macromolecules (Hunter, 2012; Kamerlin et al., 2013; Westheimer, 1977). Phosphates and orthophosphates are critical for metabolic reactions in cells, and adding or removing phosphate groups to organic molecules (sugars, nucleotides, and phospholipids) is central to metabolism (Westheimer, 1987). However, a systems-level understanding of how phosphate balance is maintained in cells, in conjunction with the many metabolic roles of phosphate, remains a poorly addressed but fundamental biological question. Building an overarching organizational logic of how phosphates are integral to and mediate metabolic information flow has therefore not been systematically attempted.

Most cells (particularly free-living microbes or sessile organisms) experience fluctuations in available nutrients. Therefore, cells have evolved multiple regulatory mechanisms to sense and adapt to nutrients. In the contexts of phosphate availability, such regulatory responses have been well studied in plants, bacteria, or model eukaryotic cells such as the budding yeast *Saccharomyces cerevisiae*. For example, in yeast, the cellular transcriptional response to extended phosphate limitation is mediated by the *PHO* pathway (Lenburg and O'Shea, 1996; Ljungdahl and Daignan-Fornier, 2012; Mouillon and Persson, 2006; Secco et al., 2012). This system functions through the transcription factors, Pho4 and Pho2, which control the expression of phosphate metabolism related genes called the *PHO* regulon. This regulon includes genes that encode high- and low-affinity phosphate transporters, acid and alkaline phosphatases, vacuolar polyphosphate (polyP) synthesis proteins and vacuolar polyphosphatase (Bun-Ya et al., 1991; Lemire et al., 1985; Martinez and Persson, 1998; Ogawa et al., 2000; To-E et al., 1973), which collectively help in the acquisition, assimilation and release of phosphate. Several such examples of elaborate transcriptional-regulatory networks that respond to phosphate starvation are also known in plants (Fabiańska et al., 2019; Stigter and Plaxton, 2015; Zhang et al., 2014), bacteria (Lamarche et al., 2008) and other organisms (Bergwitz and Jüppner, 2011).

In contrast to our extensive knowledge about regulated gene expression during phosphate starvation, how distinct nodes in cellular metabolism are themselves coupled to phosphate balance is surprisingly not as clear. Effectively, our collective understanding of phosphate demand and utilization, and the role of phosphates in regulating the metabolic state of the cell, remains poor. In this perspective, we first identify metabolic processes that are irrevocably coupled to phosphate balance, and therefore overall cellular metabolic homeostasis. We illustrate how cellular metabolism depends on intracellular phosphate amounts, and can maintain Pi levels, by effectively ‘squeezing’ internal phosphate availability through specific metabolic outputs. We conceptualize phosphates in central metabolism into cycles, sources, and sinks. By defining and distinguishing these roles, it now becomes possible to more systematically contextualize how phosphates will control biochemical information flow in cells, and thereby determine cellular metabolic states.

## Phosphates in biochemical cycles and ‘closed’ reactions

**Figure 1.**
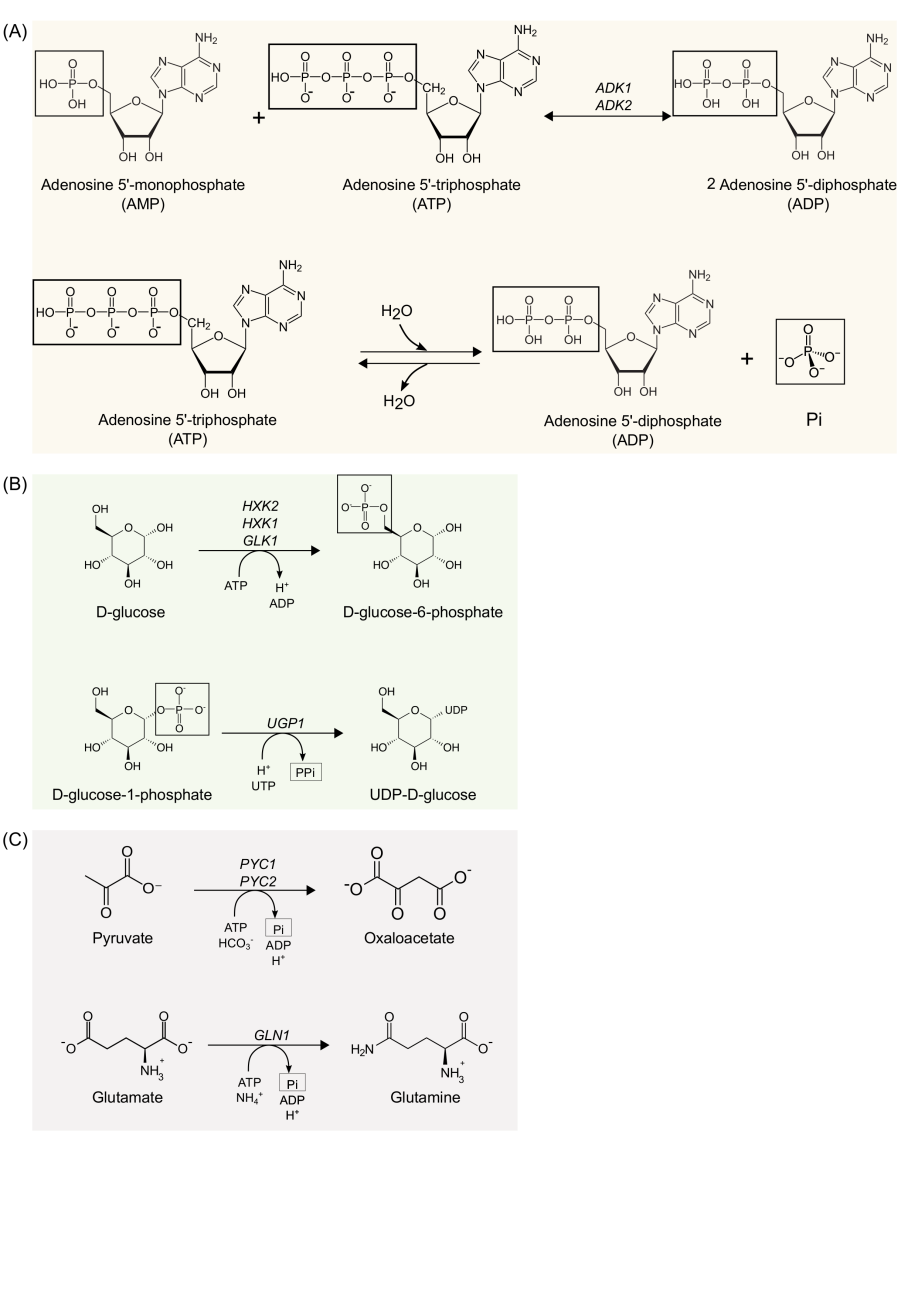
Biochemical reactions illustrating phosphate cycles in metabolism. (**A**) The synthesis and hydrolysis of ATP is depicted in the reactions. One molecule each of AMP and ATP are utilized for the synthesis of two molecules of ADP by adenylate kinase, collectively a phosphate-neutral reaction. In the second reaction, the synthesis and hydrolysis of ATP with the concurrent release or use of a Pi molecule, and a water molecule is shown. Here, the net change in Pi is zero for each cycle. (**B**) Examples where the hydrolysis of a phosphoanhydride bond drives the synthesis of phosphate-containing products. The conversion of D-glucose to D-glucose-6-phosphate by hexokinases, and D-glucose-1-phosphate to UDP-glucose by UDP-glucose pyrophosphorylase are shown. Here, ATP and UTP act as phosphate group donors, respectively. (**C**) Examples where the hydrolysis of a phosphoanhydride bond drives the synthesis of products with the release of phosphate. The conversion of pyruvate to oxaloacetate by pyruvate carboxylase, and glutamate to glutamine (by glutamine synthetase is shown). Here, the hydrolysis of a phosphoanhydride bond in ATP provides energy for the catalysis of the reaction.

Phosphates are integral components of lipids, nucleic acids, proteins, and sugars. They are present in many energetically costly metabolites, including reducing equivalents in the form of pyridine nucleotides (NADH and NADPH), cellular energy currencies (represented by adenosine triphosphate (ATP), phosphoribosyl phosphate (PRPP), pyrophosphates (PPi), pyridoxal phosphate (PLP)), and signaling molecules like inositol phosphates. Each of these molecules has substantial metabolic roles. Indeed, phosphates are ubiquitous, because of their unique chemistry. They easily ionize (and are retained within cells), yet phosphate bonds are stable, and so phosphates are ideally suited to link nucleotides, function as intermediary metabolites, and serve as energy sources (Westheimer, 1987). Under physiological conditions, many phosphate-containing metabolites participate in coupled biochemical reactions that effectively cycle the phosphate moieties (Westheimer, 1987). This type of role of phosphate-containing molecules as part of continuous cycles is relatively well-understood biochemically, and a few illustrative reactions are shown in Figure 1. Two classic examples would be ATP + AMP <->2 ADP, and the continuous cycling of ATP <-> ADP (with the concurrent release or use of a Pi molecule, and a water molecule) (Figure 1A). Other examples include reactions where hydrolysis of a phosphoanhydride bond drives product formation with either the incorporation of phosphate(s) into (Figure 1B), or the release of phosphate(s) from the product (Figure 1C).

Importantly, such instances can be viewed as contained biochemical systems. If there is a fixed amount of phosphates, no new phosphate molecules are required for such a system to continue indefinitely, *as long as the total amounts of these metabolites are unchanged*. These enclosed ‘phosphate cycles’ use phosphates integrally in their reaction mechanisms. Therefore, although these reactions require phosphates, they can be considered distinct from the metabolic reactions that can immediately affect the total phosphate pools available in the cell, or reactions that must most rapidly change when phosphates become limiting (these are discussed later). Of course, the absolute amounts of these metabolites do change, depending on the metabolic needs of the cell. By first separating out these closed phosphate cycles, we can now conceptualize how other, distinct phosphate utilizing/dependent reactions (which are not part of these phosphate cycles) can be critical for phosphate homeostasis, and consequently regulate overall carbon metabolic flux.

## Phosphate limitation and metabolic state regulation

**Figure 2.**
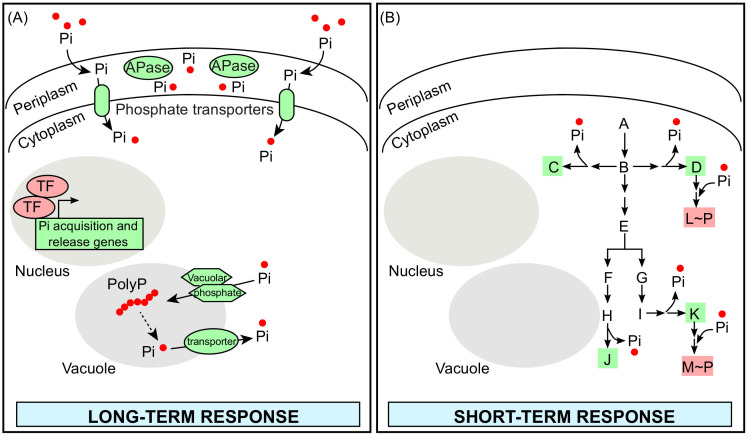
Long-term (molecular) and short-term (metabolic) responses in response to phosphate limitation. (**A**) A schematic representation of a typical long-term cellular response upon severe phosphate starvation is shown (the *S. cerevisiae PHO* system is used illustratively). This response is mediated by gene expression changes through activation of phosphate responsive transcription factors (TFs). These transcription factors increase the expression of transcripts, which encode proteins involved in phosphate acquisition and release. These include phosphate transporters, acid- and alkaline-phosphatases (APases), and vacuolar phosphate transporters. These proteins result in both the increased uptake of extracellular phosphate and the release of phosphate from intracellular phosphate pools. (**B**) A schematic representation of a hypothetical set of biochemical pathways (a toy model) is shown; illustrating how these reactions can release or consume phosphates. In such a short-term metabolic response, which is elicited upon transient fluctuations in phosphate levels, changes in metabolic flux through specific nodes can immediately restore phosphate, either through increased synthesis of molecules that will release phosphate (highlighted in green box), or by decreased synthesis of molecules that will consume phosphate (highlighted in the red box).

Phosphate limitation can be viewed as two types – (1) extreme phosphate starvation, due to a severe or continuing depletion of phosphate in the extracellular environment, or (2) transient limitations in phosphate availability and/or in intracellular Pi levels. Our understanding of transcriptional responses to extreme phosphate starvation is reasonably advanced, at least in model systems like *S. cerevisiae* and plants (Fabiańska et al., 2019; Ljungdahl and Daignan-Fornier, 2012; Secco et al., 2012; Stigter and Plaxton, 2015; Zhang et al., 2014). Here, upon phosphate starvation, a combination of increased Pi acquisition (via extracellular Pi uptake), and increased Pi mobilization (via intracellular Pi stores) enables cells to restore intracellular Pi levels. This is illustrated in Figure 2A. In budding yeast for example, both Pi acquisition and mobilization from various reserves are mediated by the activation of the *PHO* transcriptional response, which regulates gene expression of multiple phosphate transporters and acid phosphatases. However, such elaborate responses will require time scales of min–hours, involving multiple, regulated transcription and translation steps.

In contrast to severe phosphate starvation, there can also be transient limitations in internal phosphate levels. In such contexts, even before the phosphate-responsive gene-regulatory programs fully come into effect, changes in metabolism can immediately restore intracellular phosphate levels. We illustrate how this can happen, using a hypothetical reaction network (Figure 2B). In such a reaction network, some reactions will release phosphate, while others will incorporate/consume phosphate. Purely based on standard rules of mass action, when phosphate availability is reduced, the flux through reactions releasing phosphate will increase, while those that consume phosphates will decrease. Under such conditions, cells can therefore immediately mobilize Pi from intracellular metabolic reserves by biochemical processes driven by metabolite abundances and the law of mass action (Hackett et al., 2016). Thus, while regulated gene expression changes in response to severe phosphate starvation can carry out longer term adaptive responses, metabolic rewiring can rapidly maintain or restore intracellular Pi levels in response to transient fluctuations. This is the essence of dynamic metabolic regulation, where cells modulate their metabolic fluxes over short- and long-time scales to match their requirements (Metallo and Vander Heiden, 2013). Summarizing, while the long-term transcriptional, adaptive responses are well studied, we will substantiate the idea of how immediate metabolic rewiring can restore phosphates, through reactions that release or absorb Pi, stating specific examples.

A notable point emerges if such mobilization of metabolic Pi stores by mass-action based processes occur: as we will illustrate subsequently, there will be an accompanying rewiring of overall carbon flow, where reactions that release Pi are favored. This is associated with the accumulation of other central carbon (glucose) derived metabolites. This therefore highlights an underappreciated point of how tightly central carbon metabolism is coupled to phosphate homeostasis. By clearly identifying nodes of metabolism that will change with phosphate availability, we can predictably address how intracellular Pi changes will re-route carbon flux through very specific nodes of carbon metabolism to alter cellular metabolic states.

## Conceptualizing metabolic sources and sinks of phosphate

**Figure 3.**
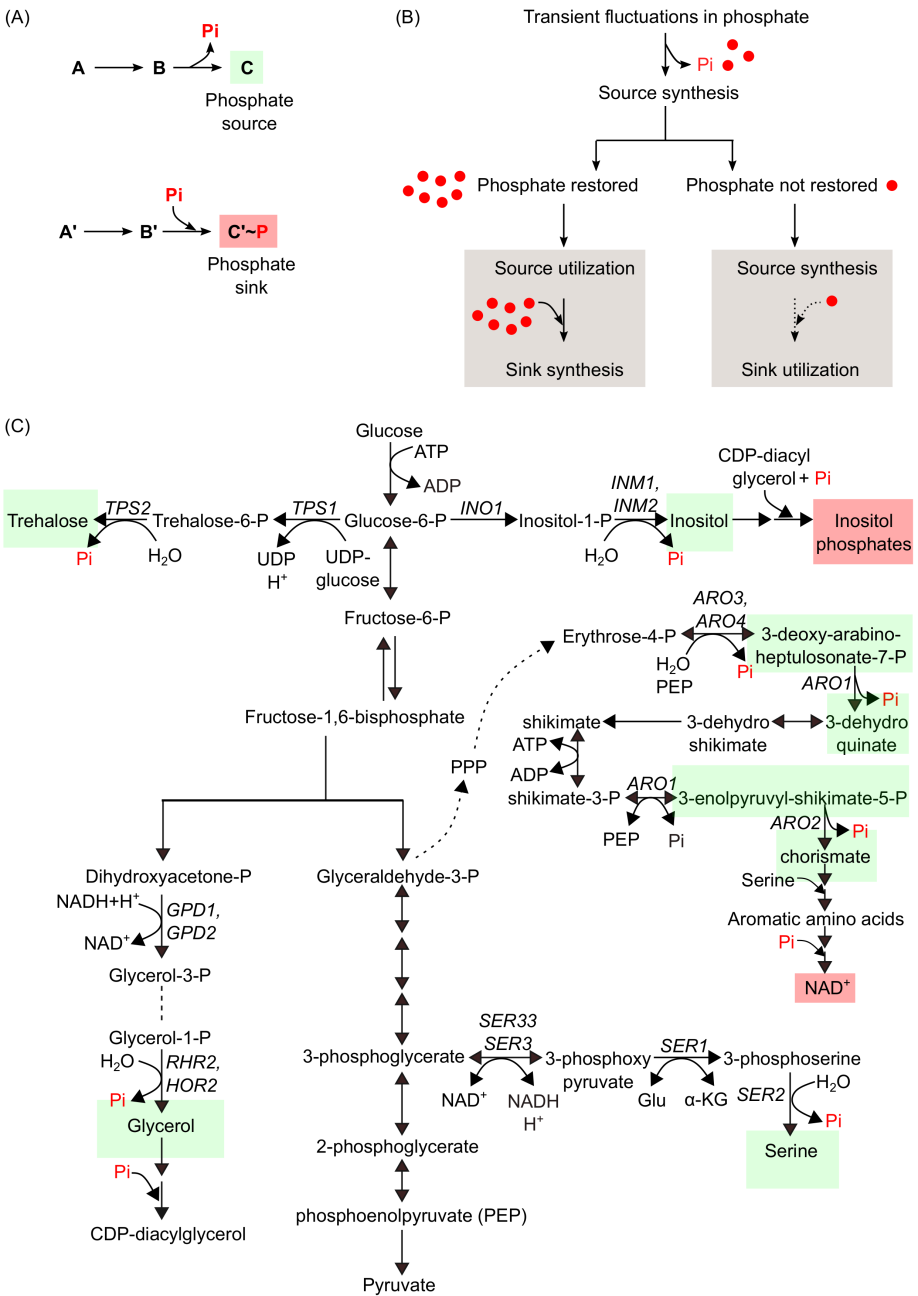
Metabolic sources and sinks of phosphates. (**A**) An illustration of a hypothetical set of biochemical reactions, representing metabolic sources and sinks of phosphates. The top reaction shows that during the synthesis of a phosphate source (highlighted in green box), phosphate is released, while the bottom reaction shows that during the synthesis of a phosphate sink (highlighted in the red box), phosphate is utilized. (**B**) A schematic showing possible outcomes that restore phosphate during transient fluctuations in phosphate availability. Through largely mass action based processes, limitations in phosphate will promote metabolic reactions leading to the synthesis of a phosphate source metabolite, accompanied with the release of Pi. As a result of this, two possible cellular scenarios can occur- first, if phosphate levels are restored through this process, this can result in the subsequent utilization of the metabolic phosphate source and phosphate for the synthesis of metabolic sink. Alternately, if source producing reactions are insufficient to restore phosphate, this can result in continuing synthesis of metabolic sources, and the possible utilization of metabolic sinks, both of which results in phosphate release. (**C**) A set of key metabolic pathways, derived from glucose metabolism, illustrating the flow of phosphates, and highlighting major reactions that result in the synthesis of metabolic phosphate sources (green boxes) accompanied by Pi release, and few reactions that result in the synthesis of metabolic phosphate sinks (red boxes) accompanied by utilization of Pi.

Extending beyond closed biochemical reactions where phosphates continuously cycle (discussed in the earlier section), we can now organize phosphate-dependent metabolic reactions into classes that would affect the internal phosphate amounts distinctly. These metabolites can be classified into two groups – phosphate sources and sinks (Figure 3A). Here, *sources* of phosphate can now be defined as metabolites which themselves may not contain phosphate/phosphorus, but importantly their synthesis results in phosphate release. These metabolites will therefore restore phosphate pools and balance Pi during transient Pi fluctuations. However, in order to do so, they should satisfy the following criteria; their synthesis should *increase* in response to phosphate limitation (and be accompanied by Pi release), the metabolic flux towards these molecules should be (conditionally) high, and they need not always accumulate in the cell. This definition therefore alters the idea of a source from a phosphate-containing molecule, to include molecules that themselves do not contain phosphates, but are made concurrent with Pi release. The metabolic precursors of these sources will be phosphate-containing molecules. As we will exemplify shortly, many metabolic pathway intermediates or ‘end products’ that do not themselves contain phosphates will act as very effective phosphate sources, since Pi will be released during the synthesis of these molecules.

Contrarily, *sinks* of phosphate will be phosphate-containing metabolites, where the synthesis of these metabolites results in the storage of excess Pi. Sinks therefore should satisfy the following criteria; the synthesis of these molecules should *decrease* in response to phosphate limitation, they should have a slow turnover rate, and should accumulate in abundance.

By using such a definition, it becomes clear that a source and a sink need not necessarily be the same molecules, and in fact may often not even be in the same biochemical pathway. However, their respective synthesis must be anti-correlated in the cell, in response to phosphate limitation. This central idea is illustrated in Figure 3B. This source-sink balance will therefore be substantially governed by the allocation and amount of intracellular Pi. In such a context, the cellular response to transient phosphate depletion can now be understood as follows: whenever there is a transient Pi depletion, increased synthesis towards a Pi source will immediately occur, and this will rapidly release phosphate (Figure 3B). If phosphate pools are not immediately restored, the phosphate sinks (which are molecules that are slowly turned over) can be further mobilized to release inorganic phosphates. In summary, simple metabolic rewiring towards the synthesis of Pi sources can initially restore Pi, and the subsequent degradation/mobilization of sinks can continue this process of Pi release. Such a definition of sources and sinks contrasts with simply phosphate-rich metabolites involved in biochemical cycles (as described earlier). Making this classification now allows a systematic investigation of their distinct roles during changes in intracellular phosphate, and this will provide a predictable metabolic state signature for the cell.

## Illustrating metabolic sources and sinks of phosphate

What therefore might be the cellular sources of phosphates, and can they now be precisely identified? We systematically investigated the central carbon derived metabolic pathways (as obtained from KEGG [Ogata et al., 1999]) to identify possible cellular phosphate sources and sinks, using an example of yeast metabolic networks. From this comprehensive analysis, trehalose, serine, chorismate, inositol, and glycerol synthesis, all of which are accompanied by the release of Pi, represent the likely examples of phosphate sources by satisfying all required criteria as defined earlier (Figure 3C). Note here that none of these metabolites – trehalose, serine, chorismate, inositol or glycerol – contain phosphates. Instead, all of these metabolites are made via intermediate reactions where Pi is released, illustrating this idea of what a Pi source might be (and that this need not be a phosphate-containing metabolite). Trehalose is a major ‘storage’ carbohydrate that accumulates in cells at very high concentrations (Chester, 1963; François and Parrou, 2001; Stewart et al., 1950), and a recent report provides strong evidence of it being a phosphate-restoration source (Gupta et al., 2019; Gupta and Laxman, 2020). Trehalose is made from the sugar phosphates, glucose-6-phosphate (G6P) and UDP-glucose in two steps, where the last step (catalyzed by Tps2) results in trehalose formation and the release of one molecule of Pi (Figure 3C). In glucose replete conditions, these two steps are involved in futile cycling of trehalose during glycolysis, and trehalose synthesis allows proper glycolytic flux by maintaining phosphate balance (van Heerden et al., 2014b; van Heerden et al., 2014a). Pertinently, dampening the *PHO* response (which mimics phosphate limitation), results in carbon (glucose) flux rerouted towards trehalose synthesis, and away from the pentose phosphate pathway (PPP) and glycolysis (Gupta et al., 2019; Gupta and Laxman, 2020). This is largely governed by mass action, with no changes in glycolytic/PPP enzyme amounts required. Multiple lines of evidence indicate that rerouting metabolic flux towards trehalose restores the Pi balance (Gupta et al., 2019). Further, if the last step in trehalose synthesis (which releases phosphate) is removed (*tps2*Δ cells), this decreases the cellular phosphate pools (Gupta et al., 2019). Taken together, all these data satisfy the criteria for trehalose to be a phosphate source. A related, predictive case can be made for glycerol, which is synthesized during the lower arm of glycolysis and is accompanied by the release of Pi. Glycerol biosynthesis from dihydroxyacetone phosphate (DHAP) occurs in two steps, where the first step results in the synthesis of glycerol-3-phosphate (catalyzed by Gpd1 and Gpd2), and the second step results in the synthesis of glycerol and the release of one Pi molecule by the action of glycerol-3-phosphatases (Hor2 and Rhr2) (Austin and Mayer, 2020; Figure 3C). In line with the role of glycerol as a phosphate source, glycerol levels are elevated in response to phosphate limitation (Kazemi Seresht et al., 2011). Moreover, consistent with a role of trehalose and glycerol syntheses in phosphate restoration, the metabolic phosphatases involved in these biochemical conversions, trehalose-6-phosphate synthase/phosphatase and glycerol-3-phosphatases, respectively, are also transcriptionally upregulated during phosphate limitation (Ogawa et al., 2000). This suggests an amplified response (through the transcriptional induction of these enzymes) as a way to mobilize metabolic reserves of Pi. Thus, when phosphates are transiently limited, glucose-6-phosphate and dihydroxyacetone phosphate will reroute away from glycolysis and the PPP, and toward trehalose and glycerol respectively. While this can restore phosphate balance, it will also result in a reduction of the overall glycolytic and PPP flux.

Three other major biosynthetic processes derived from glucose metabolism result in abundant products and also release phosphate. Serine biosynthesis from 3-phosphoglycerate (3PG) occurs in three steps, and the last step releases one Pi molecule. Chorismate synthesis (a precursor for aromatic amino acids and the vitamins, p-aminobenzoate and p-hydroxybenzoate) requires one molecule of erythrose-4-P (E4P) (from the pentose phosphate pathway) and two molecules of phosphoenolpyruvate (PEP), and occurs in seven steps. Here, three molecules of Pi are released (Figure 3C). The prediction therefore is that flux towards these molecules will increase when phosphates are limiting. Indeed, consistent with this prediction, independent studies with phosphate starvation have all observed that amino acid amounts strongly increase (Boer et al., 2010; Gupta et al., 2019), along with reduced metabolites of the PPP and glycolysis. These data therefore are consistent with these metabolites acting as phosphate sources. Finally, a predictive case can be made for myo-inositol (inositol) biosynthesis as a source of phosphate. Inositol is perhaps the most abundantly present stereoisomer in yeast and mammalian cells (Desfougères et al., 2019). Inositol biosynthesis from glucose-6-phosphate (G6P) occurs in two steps. In the first step, Ino1 converts glucose-6-phosphate to inositol-1-phosphate. The second step is catalyzed by inositol monophosphatases (Inm1 and Inm2), and inositol-1-phosphate is converted to inositol and one molecule of Pi is released (Figure 3C). Although the inositol biosynthetic pathway is relatively poorly studied in yeast, and there is insufficient metabolic data in this (Pi relevant) context, inositol monophosphatase, Inm1, is upregulated under low phosphate conditions (Kazemi Seresht et al., 2011). Therefore, we can predict that this inositol synthesis, accompanied by Pi release, can help restore internal Pi levels. To summarize, phosphate sources can modulate the overall carbon flux distribution in a metabolic network depending on the internal phosphate levels, and will thereby determine the metabolic state of the cell.

What then are the sinks of phosphates? Nucleoside-, di-, tri-phosphates, NAD(P)^+^, inositol pyrophosphates, and polyphosphates (PolyP) are all putative phosphate sinks (and not sources). Is there evidence that is consistent with the plausibility of these metabolites as phosphate sinks? Going by the earlier described criteria of a sink, these metabolites are abundant in cells (Belenky et al., 2007; Ermakova et al., 1981; Gakière et al., 2018; Kukko and Saarento, 1983; Ljungdahl and Daignan-Fornier, 2012; Pestov et al., 2004; Rao et al., 1998). A second criterion is that upon phosphate limitation, their levels will reduce due to both decreased synthesis and increased degradation (mobilization), and this will release phosphate. The case for nucleotides as phosphate sinks: as sinks, the synthesis of these molecules should *decrease* in response to phosphate limitation, they should have a slow turnover rate, and should accumulate in abundance. These criteria appear to be well-satisfied for nucleotides (mono-, di-, tri phosphates). Phosphate limitation results in a rapid decrease in both their synthesis and steady-state levels (Ashihara et al., 1988; Boer et al., 2010; Klosinska et al., 2011). In a recent study, the reduction in (but not absence of) *PHO* gene expression (phosphate transporters, phosphatases etc.), which mimics phosphate limitation, also revealed reduced de novo nucleotide synthesis. This was not due to reduced amounts of nucleotide biosynthesis enzymes, but instead was governed by mass action (Gupta et al., 2019). Further, phosphate limitation also increases the amounts of 5’-, 3’-nucleotidases and nucleotide pyrophosphatase enzymes, which thereby increases nucleotide degradation (and will allow phosphate release) (Alipanah et al., 2018; Hammond et al., 2003; Kennedy et al., 2005; Misson et al., 2005; Rittmann et al., 2005; Shimano and Ashihara, 2006; Uhde-Stone et al., 2003; Wasaki et al., 2006; Yin et al., 2007). Phm8, a nucleotidase, is transcriptionally induced (a longer term response) upon carbon, nitrogen, and Pi starvation (Xu et al., 2013). The relavance of these nucleotidases (in the context of Pi sinks) will become clearer in the following paragraph.

Using very similar analyses, the phosphate moiety of NAD(P)^+^ and its intermediate metabolites also become potential phosphate sinks. Although direct estimates of these molecules during phosphate limitation have not been made, we can predict that there will be reduced synthesis of these metabolites, as they utilize nucleotide triphosphates as their phosphate donor, and nucleotide synthesis itself decreases under these conditions. Indeed, consistent with this prediction, during low Pi conditions, pyridine nucleotides, NAD^+^ and NAD^+^ intermediates (NMN and NaMN) are broken down by specific nucleotidases, and this will also release phosphate (Bogan and Brenner, 2010). Indeed, somewhat overlooked reports suggest connections between phosphate signaling and NAD(P)^+^ metabolism (Bogan and Brenner, 2010; Kato and Lin, 2014).

In this context of nucleotides, as well as NAD(P)^+^ molecules as Pi sinks, two relevant points emerge. In an insightful review, Bogan and Brenner summarize that ribonucleoside monophosphates (which are potential substrates of 5’-nucleotidases) come from both salvage and de novo synthesis. The 5’-nucleotidases themselves allow the reverse reactions of nucleoside kinases (and therefore oppose salvage pathways, and nucleotide generation) (Bogan and Brenner, 2010). These opposing substrate cycles, and the relative activity of phosphorylation/dephosphorylation, determine how much nucleoside monophosphates (and eventually triphosphates) are present, or if they will be converted to nucleosides. A more recent review points to the fact that upon extreme Pi starvation, as a ‘last resort’ cells induce enzymes that release Pi from nucleotides, since it would otherwise be illogical for cells to deplete its pools of nucleotides, which are critical for future cell division (Austin and Mayer, 2020). These points are all entirely consistent with nucleotides (and NAD related molecules) being Pi sinks, which will serve to eventually release Pi if phosphate levels within cells are not restored.

Finally, inositol pyrophosphates and polyphosphates are likely phosphate sink candidates. Inositol pyrophosphates have the highest proportion of phosphate groups for any metabolite, containing more phosphates than carbon atoms (Saiardi, 2012), and they regulate the expression of *PHO* related transcripts in yeast (Auesukaree et al., 2005; Lee et al., 2008; Lee et al., 2007), suggesting cross-talk between inositol pyrophosphate metabolism and phosphate signaling. Polyphosphates (polyP) are linear polymers of many phosphate residues, ranging from few to hundreds, and are linked by the same high-energy phosphoanhydride bonds that are found in ATP. PolyPs are mainly stored inside the vacuole, with small pools also found in cytosol, nucleus and mitochondria (Gerasimaitė and Mayer, 2016; Saiardi, 2012). Evidence from some organisms (bacteria, yeasts) suggest that polyphosphates are effective phosphate reservoirs, and upon phosphate starvation, these stores are degraded by the action of exo- and endo-polyphosphatases to restore phosphate (Brown and Kornberg, 2008; Kulaev et al., 1999; Kulaev and Kulakovskaya, 2000; Shirahama et al., 1996). We would therefore predict these metabolites to collectively be intracellular phosphate sinks, with inositol phosphates and polyphosphates likely to be the major sinks. All these are now directly testable predictions.

Two broad points emerge when we define phosphate sources and sinks in this way. First, we reiterate a close coupling of glucose metabolism with internal phosphate homeostasis. Altering flux toward different arms of glucose metabolism – glycolysis, the pentose phosphate pathway, and trehalose/glycogen synthesis – will therefore result in substantial changes in phosphate release/utilization (as shown earlier in Figure 3C). Second, we can now with some confidence predict key metabolic state changes (based on metabolic flux through these defined sources and sinks) due to a transient phosphate limitation (or ‘squeeze’). Primarily, trehalose, aromatic amino acids/chorismate, serine, glycerol, and inositol (phosphate sources) will all increase, while nucleotides, polyphosphates and (some) inositol pyrophosphates (phosphate sinks) will decrease. Flux through glycolysis and the pentose phosphate pathway will correspondingly decrease. Amounts of these hallmark metabolites therefore represent a concise metabolic signature of a phosphate-limited cell. In summary, this conceptualization of the phosphate sources and sinks together with their biochemical reactions illustrate the importance of these metabolites in maintaining the overall phosphate levels in the cell. Second, mass-action driven metabolic processes play a fundamental role in phosphate homeostasis by regulating flux through the key phosphate-related metabolic nodes. Collective evidence therefore indicates that there is an intimate relationship and interdependence between phosphate balance and carbon metabolism.

## A final challenge: to understand distinct phosphate pools and their roles in metabolic homeostasis

While bacteria do not have internal pools of phosphates, in eukaryotic cells, phosphates reside in multiple cellular compartments, including the cytoplasm, nucleus, vacuole, endoplasmic reticulum, mitochondria and so on (Beauvoit et al., 1989; Kato and Lin, 2014). Phosphate supply inside the cell therefore depends on the transport of phosphate across the plasma membrane and membranes of various organelles mediated by transporter proteins. The content and functions of phosphates vary in different organelles. Inorganic phosphate is required for the production of ATP in both the cytosol and mitochondria. In the cytosol, Pi stimulates the activity of hexokinase and phosphofructokinase, which catalyze irreversible steps of glycolysis. Moreover, Pi is a necessary substrate for glyceraldehyde-3-phosphate dehydrogenase, which catalyzes the first reaction of the payoff phase in glycolytic pathway (and therefore is a major consumer of phosphate). In mitochondria, inorganic phosphate is required for oxidative phosphorylation, as the ATP synthase complex uses it for producing ATP (Ferreira and Pedersen, 1993). Vacuoles have high amounts of inorganic phosphates in the cell. Estimates of the Pi concentration in vacuoles is ~100 mM, which is ~100 times higher than cytosolic concentrations (Okorokov et al., 1980). The mitochondrial polyPs, which are 14 residues long, constitute ~10% of the total cellular polyP content (Beauvoit et al., 1989). In yeast, cell envelopes also contain polyP, with ≥20% of the total cellular polyP content (Kulaev and Kulakovskaya, 2000). Vacuoles are the major polyP storage compartments in yeast, with the total polyP concentration of ~200 mM (Kornberg, 1999; Urech et al., 1978). By increasing the mobilization and transport of phosphate into the cytosol, vacuolar polyP helps maintain cellular phosphate homeostasis during limiting-phosphate conditions. A genetic screen in yeast revealed the distribution of proteins important for polyP content in various compartments: cytoplasm (59 proteins), nucleus (56 proteins), mitochondria (42 proteins), vacuole (20 proteins), and endoplasmic reticulum (13 proteins), collectively indicating that multiple groups of proteins help manage polyP metabolism in distinct cellular compartments (Freimoser et al., 2006). In line with this, two different vacuolar transport sub-complexes: Vtc4/Vtc3/Vtc1 and Vtc4/Vtc2/Vtc1 are present in the cell, the first on the vacuolar membrane and the second in endoplasmic reticulum and nucleus (Gerasimaitė and Mayer, 2016). Together, these studies suggest that cellular phosphate pools have distinct subcellular localizations. This adds additional (and currently entirely unaddressed) complexity in how phosphate homeostasis is maintained in the cell. We know relatively little about how intracellular pools of phosphates control metabolic information flow. Comprehensively addressing the role of phosphates in mediating metabolic information flow will therefore require such knowledge of how phosphates in these distinct pools function in unison.

## Concluding remarks

Surprisingly, an integrative understanding of phosphates as central regulators of metabolic information transfer in cells has been missing. Because phosphates are everywhere, this appears to be an intractable problem. However, by going beyond investigating molecular responses to phosphate limitation, and by accounting for and systematically reorganizing both short- and long-term responses (mediated by metabolic/mass action and molecular control respectively), we can obtain insights into coordinated process that maintain phosphate levels. In this article, we have conceptualized and categorized phosphates into metabolic cycles, and then sinks and source. This is a first step toward building an integrative understanding of how phosphates control metabolic information flow. Since the metabolic control of phosphate homeostasis depends on central carbon metabolism and vice versa, identifying control-hubs that facilitate cross-talk between these two systems should therefore be an exciting area of future inquiry.
